# Knowledge, attitudes, and practices of hand eczema patients in Guangdong, China

**DOI:** 10.3389/fpubh.2025.1706796

**Published:** 2025-12-18

**Authors:** Jiecheng Zheng, Shaoling Zhao, Xueyi Huang, Xin Yao, Qing Chen, Min Tan, Jia Liao

**Affiliations:** 1 Department of Dermatology, Zhongshan Second People's Hospital, Zhongshan, Guangdong, China; 2 School of Humanities and Social Science, Zhongshan Institute, University of Electronic Science and Technology of China, Zhongshan, Guangdong, China

**Keywords:** hand eczema, knowledge, attitudes, practices, cross-sectional study

## Abstract

**Background:**

Hand eczema is a common, debilitating condition affecting quality of life. This study aimed to assess the knowledge, attitudes, and practices (KAP) of patients with hand eczema in Guangdong, China, toward their condition.

**Methods:**

A cross-sectional study was conducted at our hospital from September 21, 2023, to July 31, 2024, using a self-designed KAP questionnaire.

**Results:**

A total of 612 valid questionnaires were collected. The mean (SD) scores for knowledge, attitude, and practice were 9.25 (2.55) (possible range: 0–23), 35.68 (6.02) (possible range: 10–50), and 24.03 (5.18) (possible range: 8–40), respectively. Multivariate logistic regression indicated that a higher attitude score (OR = 1.209, *p* < 0.001) was independently associated with more proactive practices. The path analysis revealed that knowledge directly influenced attitude (*β* = 0.191, *p* < 0.001), and attitude had a direct effect on practice (*β* = 0.591, *p* < 0.001). Additionally, knowledge indirectly affected practice through attitude (*β* = 0.113, *p* < 0.001).

**Conclusion:**

Our findings highlight a significant gap in patient knowledge, suggesting that targeted educational interventions may be a valuable strategy to improve self-management. Future research should test the efficacy of such interventions.

## Introduction

Hand eczema, also known as dermatitis, is one of the most common types of eczema, primarily affecting the palms but also other parts of the hand. It is a prevalent condition, with a 1-year prevalence of up to 10% in the general population (1). In China, recent studies have reported a prevalence ranging from 8.8 to 12.3% in the general population, with higher rates observed in certain occupational groups (1). The main symptoms include dry, itchy skin, which may appear red or darker than the surrounding skin. Additional symptoms include cracking, soreness, and bleeding (2, 3), and in some cases, blisters may form. The affected skin often becomes dry, scaly, and thickened, and during flare-ups, the fingers can swell. This condition severely impacts patients’ quality of life, placing a significant burden on patients, both psychosocially and economically (4). In more severe cases, chronic hand eczema can cause substantial pain, restricting patients’ ability to perform daily activities, making it difficult for patients to perform everyday tasks like buttoning clothes, holding a pen, or using a computer (5, 6). Hand eczema can arise from various causes, such as irritant or allergic contact dermatitis (7, 8). Treatments for hand eczema include a variety of interventions, such as avoiding known triggers, using emollients, topical corticosteroids (TCS), and phototherapy (9). Alongside these treatments, it is equally important to avoid known irritants and allergens and to consistently use moisturizers, with the primary responsibility for their daily management resting with the patient. However, despite clear treatment guidelines and preventive strategies, the clinical management of hand eczema still faces substantial challenges in reality. Many patients experience recurrent flares and persistent impairment in quality of life. This indicates that therapeutic regimens alone, such as topical corticosteroids, are insufficient. Instead, patient self-management behaviors and adherence—the “Practice” component—may represent a critical bottleneck in improving disease outcomes. Inadequate patient knowledge, negative attitudes, or poor behavioral compliance could contribute to persistent disease activity (10).

The Knowledge, Attitude, and Practice (KAP) model posits that individual behaviors are shaped by one’s knowledge and attitudes. This model is particularly relevant in chronic disease management (11, 12). Given the complexity of managing hand eczema and the potential for mismanagement or inadequate self-care, understanding patients’ KAP is essential for identifying knowledge and behavioral gaps that may impede effective treatment. Patients with hand eczema often demonstrate inadequate knowledge about their condition and its management, with one study finding that only 45% of patients could correctly identify common triggers (13). Previous studies in this field have been limited; for example, one study (14) focused solely on knowledge without addressing attitudes or practices. A broader review of the existing literature also indicates that most studies have not comprehensively evaluated all components of KAP (13). Based on the KAP model, we hypothesized that higher knowledge scores would be positively associated with more positive attitudes, which in turn would predict more proactive self-management practices among patients with hand eczema. This study aimed to test these relationships using a cross-sectional design and path analysis.

## Materials and methods

### Study design and participants

This cross-sectional study was conducted at our hospital from September 21, 2023, to July 31, 2024, focusing on patients diagnosed with hand eczema. This study was approved by the Ethic Committee of Zhongshan Second People’s Hospital (Approval Number: 2024051). Informed consent was obtained electronically from participants before data collection.

The inclusion criteria required participants to be 18 years or older and to have a clinical diagnosis of hand eczema, as defined by the 2021 Chinese Expert Consensus on Diagnosis and Treatment of Hand Eczema. Exclusion criteria included patients under 18 years of age, individuals with eczema in areas other than the hands, and those unable to complete the questionnaire.

The research team, comprising seven doctors, underwent standardized training to ensure consistent data collection. Patients attending the dermatology outpatient department were recruited through convenience sampling, and questionnaires were distributed both online and as paper versions. The Hand Eczema Severity Index (HECSI) scores were assessed by doctors or research assistants, while patients independently completed the remaining parts of the questionnaire. To maintain data quality, each patient was only allowed to submit the questionnaire once, and all questions were mandatory. The research team reviewed each questionnaire for completeness, internal consistency, and coherence, discarding incomplete or contradictory responses. Of the 650 questionnaires distributed, 638 were collected, with 612 valid responses remaining after excluding those from participants under 18, incomplete questionnaires, and those with mutually exclusive answers.

### Questionnaire introduction

The questionnaire was designed and refined with input from five dermatology specialists and one sociology expert. Following the development of the initial draft, two pilot studies involving 32 participants were conducted to test the questionnaire, resulting in a Cronbach’s *α* of 0.843, indicating strong internal consistency. The final version of the questionnaire is composed of four sections: demographic information (including gender, age, education level, place of residence, occupation, years of work, medical history, etc.), knowledge dimension (focusing on general knowledge of hand eczema, identification of causes, and prevention and treatment), attitude dimension toward hand eczema care, and practice dimension of hand eczema care. The full questionnaire and scoring are provided in the Appendix. Following the practice in similar KAP studies, a score below 70% of the maximum possible score was operationally defined as ‘insufficient’ for knowledge, attitude, and practice dimensions. A score of 70% or higher in any section was considered to indicate adequate knowledge (>16), a positive attitude (>35), and proactive practices (>28) toward hand eczema care (15, 16).

### Severity and quality of life assessment

This study used the HECSI and the Dermatology Life Quality Index (DLQI) to assess patients’ clinical severity and quality of life. The HECSI score was evaluated by the research physicians based on clinical presentation and was categorized as follows: “almost clear” (1–16 points), “moderate” (17–37 points), and “severe” (38–116 points). The DLQI questionnaire was self-completed by the patients. The DLQI scoring criteria were interpreted as: “no or minor effect” (0–5 points), “moderate effect” (6–10 points), “severe effect” (11–20 points), and “very severe effect” (21–30 points).

### Sample size calculation

The sample size was calculated based on the formula for a single population proportion, assuming a proportion (p) of 0.5 to maximize sample size, a type I error (*α*) of 0.05, and a standard error (*δ*) of 0.05. Assuming an effective questionnaire recovery rate of 90%, the final target is to collect at least 430 completed questionnaires.

### Statistical methods

Data analysis was conducted using Stata 18.0 (Stata Corporation, College Station, TX, USA). Descriptive statistics [means ± SD for normal data; medians (IQR) for non-normal data; frequencies (%) for categorical data] were calculated. Group comparisons used t-tests (normal, 2 groups), Wilcoxon-Mann–Whitney tests (non-normal, 2 groups), ANOVA (normal, ≥3 groups), and Kruskal-Wallis tests (non-normal, ≥3 groups). Correlations were assessed using Pearson (normal) or Spearman (non-normal) coefficients. Regression analyses (univariate and multivariate) explored relationships between demographic variables and dimension scores (dependent variables), with variables showing *p* < 0.1 in univariate analysis included in multivariate models. For logistic regression analyses, dimension scores were dichotomized based on their median values. Statistical significance was set at *p* < 0.05, with *p*-values reported to three decimal places. Path analysis within the KAP model examined attitude mediation between knowledge and practice, quantifying direct and indirect effects.

## Results

### Questionnaire quality assessment

A total of 612 valid questionnaires were collected. The overall scale and subscales demonstrated good internal consistency, with a Cronbach’s *α* coefficient of 0.8207 for the entire scale. The Kaiser-Meyer-Olkin (KMO) value for the overall scale was 0.8835, indicating the adequacy of the sample for factor analysis. Confirmatory factor analysis supported the three-dimensional structure of the questionnaire [root mean square error of approximation (RMSEA) = 0.047, standardized root mean square residual (SRMR) = 0.052, Tucker–Lewis index (TLI) = 0.814, comparative fit index (CFI) = 0.828], indicating acceptable model fit (Supplementary Figure S1).

### Basic information of the respondents

Of the 612 respondents, 413 (67.5%) were female, 352 (57.5%) had a college or bachelor’s degree, and 414 (67.6%) had been employed for more than 5 years. Additionally, 513 (83.8%) resided in urban areas, while 99 (16.2%) were from rural areas. Furthermore, 241 (39.4%) had experienced hand eczema for 3 months to 1 year, 246 (40.2%) had a DLQI score indicating a severe impact, and 301 (49.2%) had a HECSI score indicating moderate severity (Table 1).

**Table 1:** Demographic characteristics and KAP scores.

*N* = 612	*N* (%)	Knowledge	*p*	Attitude	*p*	Practice	*p*
		Median [25,75%] or mean (SD)		Median [25,75%] or mean (SD)		Median [25,75%] or mean (SD)	
Total score	612 (100.0)	9.25 (2.55)		35.68 (6.02)		24.03 (5.18)	
Gender			0.621		0.716		0.570
Male	199 (32.5)	9.26 (2.77)		35.86 (5.82)		24.15 (5.47)	
Female	413 (67.5)	9.24 (2.44)		35.60 (6.12)		23.98 (5.03)	
Age			0.088		0.454		**0.033**
30 years old or below	122 (19.9)	9.63 (2.81)		35.72 (5.94)		23.82 (5.30)	
31–35 years old	156 (25.5)	9.31 (2.09)		36.21 (6.11)		24.87 (4.93)	
36–40 years old	161 (26.3)	9.40 (2.19)		35.71 (5.96)		24.34 (5.06)	
40 years old or above	173 (28.3)	8.78 (2.97)		35.16 (6.06)		23.14 (5.30)	
Education			**0.003**		0.339		**0.006**
Middle school and below	54 (8.8)	7.74 (3.08)		34.30 (6.43)		21.65 (5.22)	
High school/technical school	172 (28.1)	9.20 (2.49)		35.93 (5.82)		24.19 (5.35)	
College/bachelor’s degree	352 (57.5)	9.45 (2.45)		35.72 (6.12)		24.24 (5.06)	
Master’s degree and above	34 (5.6)	9.79 (2.21)		36.24 (5.18)		24.91 (4.54)	
Residence			**0.011**		0.215		**0.005**
Urban	513 (83.8)	9.37 (2.38)		35.81 (5.99)		24.26 (5.08)	
Rural	99 (16.2)	8.61 (3.22)		35.02 (6.19)		22.84 (5.52)	
Occupation			0.301		0.424		**0.002**
Healthcare worker	24 (3.9)	10.33 (2.32)		37.75 (6.15)		25.96 (5.03)	
Beauty and hairdressing industry	28 (4.6)	8.50 (2.19)		34.29 (6.76)		23.71 (5.78)	
Housewife	121 (19.8)	9.30 (2.69)		35.20 (6.48)		24.36 (5.40)	
Cleaning and janitorial work	108 (17.6)	9.14 (2.03)		35.18 (5.78)		23.73 (4.49)	
Hardware/construction industry	88 (14.4)	9.02 (2.52)		36.34 (5.25)		23.85 (5.36)	
Food service industry	142 (23.2)	9.32 (1.83)		35.89 (5.79)		24.71 (4.76)	
Clerical staff	77 (12.6)	9.05 (2.71)		35.25 (5.73)		23.64 (5.11)	
Student/Other	24 (3.9)	10.38 (5.69)		37.71 (7.94)		20.04 (6.48)	
Years of employment			0.740		0.947		0.982
≤2 years	79 (12.9)	9.20 (2.13)		35.59 (5.07)		24.10 (4.82)	
2–5 years	119 (19.4)	9.44 (2.55)		35.63 (6.45)		24.13 (5.29)	
>5 years	414 (67.6)	9.21 (2.63)		35.71 (6.08)		23.99 (5.22)	
Duration of Hand Eczema			0.884		0.845		0.786
≤3 months	160 (26.1)	9.29 (2.31)		35.56 (5.63)		23.95 (4.54)	
3 month-1 year	241 (39.4)	9.30 (2.57)		35.83 (6.15)		24.30 (5.37)	
1–2 years	119 (19.4)	9.29 (2.52)		35.70 (6.50)		24.06 (5.59)	
>2 years	47 (7.7)	9.00 (2.95)		35.49 (5.80)		23.42 (5.17)	
Dermatology Life Quality Index (DLQI) Score			0.457		0.803		0.776
No or mild impact (0–5 points)	160 (26.1)	9.27 (2.73)		36.19 (5.46)		24.00 (4.92)	
Moderate impact (6–10 points)	165 (27.0)	9.13 (2.91)		35.61 (6.30)		24.08 (5.60)	
Severe impact (11–20 points)	246 (40.2)	9.38 (2.19)		35.45 (6.24)		24.14 (5.14)	
Very severe impact (21–30 points)	41 (6.7)	8.85 (2.30)		35.41 (5.71)		23.29 (4.69)	
Hand Eczema Severity Index (HECSI) Score			0.756		0.521		0.258
Almost clear, 1–16	75 (12.3)	9.51 (3.12)		36.20 (6.20)		23.80 (4.89)	
Moderate, 17–37	301 (49.2)	9.18 (2.66)		35.91 (5.75)		23.78 (5.44)	
Severe, 38–116	236 (38.6)	9.26 (2.20)		35.23 (6.29)		24.43 (4.91)	
Have you or your immediate family members ever had a history of atopic diseases?			0.132		0.138		0.223
Yes	57 (9.3)	9.40 (3.52)		34.26 (6.81)		23.12 (5.22)	
No	262 (42.8)	9.08 (2.88)		36.05 (6.08)		23.92 (5.60)	
Not sure	293 (47.9)	9.38 (1.95)		35.63 (5.78)		24.30 (4.75)	
Have you ever undergone a patch test (allergen test)?			0.168		0.439		0.175
Yes	54 (8.8)	9.59 (3.01)		36.48 (7.17)		25.15 (5.68)	
No	418 (68.4)	9.13 (2.62)		35.62 (5.99)		23.85 (5.27)	
Not sure	139 (22.7)	9.50 (2.08)		35.58 (5.65)		24.17 (4.64)	

Bold values indicate a statistically significant difference (*P* < 0.05).

### Knowledge, attitudes, and practices

The mean (SD) scores for knowledge, attitude, and practice were 9.25 (2.55), 35.68 (6.02), and 24.03 (5.18), respectively. The mean knowledge score was 9.25/23 (40.2%), which falls below the pre-defined threshold for sufficiency. The mean practice score was 24.03/40 (60.1%), also below the sufficiency threshold, while the mean attitude score was 35.68/50 (71.4%), indicating a positive attitude. Patients with different educational levels showed differences in their knowledge (*p* = 0.003) and practice (*p* = 0.006) scores. Urban residents had higher knowledge (*p* = 0.011) and practice (*p* = 0.005) scores compared to rural residents. Additionally, the practice scores of patients with different occupations differed (Table 1). Notably, no statistically significant associations were observed between disease severity (HECSI) or quality of life impairment (DLQI) and patients’ attitude scores (*p* = 0.521 and *p* = 0.803, respectively; see Table 1).

In the knowledge dimension, key areas of uncertainty included the appropriate use of gloves (K11, 342/612, 55.9% “Unclear”), the universal need for patch testing (K7, 318/612, 52.0%), and the role of topical corticosteroids as a first-choice treatment (K10, 278/612, 45.4%)(Table 2). In the attitudinal dimension, (321/612, 52.5%) disagreed and (53/612, 8.7%) strongly disagreed that changing jobs could alleviate severe cases of hand eczema (A10), 80 (13.1%) disagreed and 11 (1.8%) strongly disagreed that wearing rings and other hand jewelry negatively impacts eczema (A7); and 71 (11.6%) disagreed and 3 (0.5%) strongly disagreed that applying moisturizer in addition to using gloves can enhance protective effects (A6) (Table 3). Regarding the practice dimension, (468/612, 76.5%) did not (inconsistent or very inconsistent) wear gloves outdoors in cool weather to protect hands from drying out (P8); 336 (54.9%) did not (inconsistent or very inconsistent) apply moisturizer multiple times after finishing work each day and before bedtime (P4); and 334 (54.6%) did not (inconsistent or very inconsistent) wear cotton gloves underneath occlusive gloves when worn for more than 10 min (P3) (Table 4).

**Table 2:** Distribution of knowledge dimension responses.

Knowledge	True	False	Not sure
1. Hand eczema is an inflammatory skin disease that occurs on the hands.	443 (72.4%)	47 (7.7%)	122 (19.9%)
2. Hand eczema is always accompanied by itching.	327 (53.4%)	199 (32.5%)	86 (14.1%)
3. Hand eczema is characterized by diverse clinical manifestations, complex etiology, prolonged course, and difficulty in treatment.	543 (88.7%)	25 (4.1%)	44 (7.2%)
4. Individuals with an atopic constitution (allergic constitution) have a significantly higher risk of developing hand eczema compared to those without an atopic constitution.	339 (55.4%)	51 (8.3%)	222 (36.3%)
5. The etiology of hand eczema is complex. External causes may include: (Multiple choices allowed) Metal products (nickel, chromium, cobalt)	209 (34.2%)	403 (65.8%)	
Natural rubber	192 (31.4%)	420 (68.6%)	
Fragrances	208 (34%)	404 (66%)	
Food proteins (raw meat, animal organs, grains)	221 (36.1%)	391 (63.9%)	
Strong irritants such as acids, alkalis, organic solvents, or other chemical products	174 (28.4%)	438 (71.6%)	
Weak irritants such as water, soap, detergents, oil, printing ink	173 (28.3%)	439 (71.7%)	
Mechanical damage, such as trauma, scratching, prolonged friction	128 (20.9%)	484 (79.1%)	
None of the above	16 (2.6%)	596 (97.4%)	
6. The etiology of hand eczema is complex. Internal causes may include: (Multiple choices allowed) Genetic factors	238 (38.9%)	374 (61.1%)	
Atopic constitution	227 (37.1%)	385 (62.9%)	
Mental state	231 (37.7%)	381 (62.3%)	
Hormonal levels	186 (30.4%)	426 (69.6%)	
Immune status of the body	203 (33.2%)	409 (66.8%)	
Changes in trace elements	120 (19.6%)	492 (80.4%)	
None of the above	26 (4.2%)	586 (95.8%)	
7. To identify potential allergens, all patients with hand eczema undergo patch testing (allergen testing).	252 (41.2%)	42 (6.9%)	318 (52%)
8. Damage to the skin barrier function is the central factor in the recurrence of hand eczema.	455 (74.3%)	47 (7.7%)	110 (18%)
9. When washing hands, patients with hand eczema should avoid using soaps or other cleansers. Instead, they should rinse their hands with warm water, thoroughly dry them, and promptly moisturize.	336 (54.9%)	152 (24.8%)	124 (20.3%)
10. Topical corticosteroids are the first choice of treatment for patients with hand eczema (e.g., hydrocortisone cream, desonide cream, mometasone furoate cream).	283 (46.2%)	51 (8.3%)	278 (45.4%)
11. Patients with hand eczema should preferably use rubber gloves (nitrile gloves) and film gloves (vinyl gloves).	215 (35.1%)	55 (9%)	342 (55.9%)
12. If gloves are not worn, moisturizers should be applied frequently when working in wet environments or with wet hands.	229 (37.4%)	86 (14.1%)	297 (48.5%)

**Table 3:** Distribution of attitude dimension responses.

Attitude	Strongly agree	Agree	Neutral	Disagree	Strongly disagree
1. Frequent handwashing should be avoided.	94 (15.4%)	263 (43%)	180 (29.4%)	71 (11.6%)	4 (0.7%)
2. Direct contact with chemical products such as hand sanitizers, laundry detergents, and alcohol should be avoided.	108 (17.6%)	272 (44.4%)	164 (26.8%)	62 (10.1%)	6 (1%)
3. Moisturizers should be used frequently and consistently.	96 (15.7%)	296 (48.4%)	151 (24.7%)	63 (10.3%)	6 (1%)
4. Protective gloves should be worn when working in wet environments.	91 (14.9%)	306 (50%)	143 (23.4%)	61 (10%)	11 (1.8%)
5. The material and usage of gloves are crucial.	110 (18%)	284 (46.4%)	154 (25.2%)	58 (9.5%)	6 (1%)
6. Applying moisturizer in addition to using gloves can enhance protective effects.	87 (14.2%)	270 (44.1%)	181 (29.6%)	71 (11.6%)	3 (0.5%)
7. Wearing rings and other hand jewelry negatively impacts eczema.	98 (16%)	252 (41.2%)	171 (27.9%)	80 (13.1%)	11 (1.8%)
8. Avoid vigorous scratching or rubbing of the hands.	112 (18.3%)	287 (46.9%)	156 (25.5%)	50 (8.2%)	7 (1.1%)
9. Actively identifying and eliminating any potential causes and aggravating factors is important for treating hand eczema.	126 (20.6%)	303 (49.5%)	135 (22.1%)	42 (6.9%)	6 (1%)
10. Changing jobs can alleviate severe cases of hand eczema.	63 (10.3%)	80 (13.1%)	95 (15.5%)	321 (52.5%)	53 (8.7%)

**Table 4:** Distribution of practice dimension responses.

Practice	Very consistent	Relatively consistent	Neutral	Relatively inconsistent	Very inconsistent
1. Use fragrance-free topical skincare and moisturizing products (e.g., hand cream).	82 (13.4%)	75 (12.3%)	147 (24%)	242 (39.5%)	66 (10.8%)
2. Do not wear rings or other jewelry when working with wet hands.	126 (20.6%)	235 (38.4%)	163 (26.6%)	51 (8.3%)	37 (6%)
3. When wearing occlusive gloves for more than 10 min, wear cotton gloves underneath.	90 (14.7%)	82 (13.4%)	106 (17.3%)	105 (17.2%)	229 (37.4%)
4. Apply moisturizer multiple times after finishing work each day and before bedtime.	91 (14.9%)	56 (9.2%)	129 (21.1%)	239 (39.1%)	97 (15.8%)
5. Do not reuse disposable gloves.	178 (29.1%)	293 (47.9%)	74 (12.1%)	41 (6.7%)	26 (4.2%)
6. Wash hands with cold/warm water, avoiding hot water.	159 (26%)	259 (42.3%)	121 (19.8%)	61 (10%)	12 (2%)
7. Evenly apply moisturizer to the entire hand, including fingertips, between fingers, and the back of the hands.	85 (13.9%)	65 (10.6%)	136 (22.2%)	291 (47.5%)	35 (5.7%)
8. Wear gloves outdoors in cool weather to protect your hands from drying out.	46 (7.5%)	37 (6%)	61 (10%)	173 (28.3%)	295 (48.2%)

### Correlation analysis of knowledge, attitudes, and practices

Knowledge and attitude were correlated (*r* = 0.143, *p* < 0.001), as were knowledge and practice (*r* = 0.145, *p* < 0.001). Attitude and practice showed a moderate correlation (*r* = 0.611, *p* < 0.001) (Table 5).

**Table 5:** Correlation analysis.

Spearman	Knowledge dimension	Attitude	Practice
Knowledge dimension	1.000		
Attitude	0.143 (*p* < 0.001)	1.000	
Practice	0.145 (*p* < 0.001)	0.611 (*p* < 0.001)	1.000

### Univariate and multivariate analysis of knowledge, attitudes, and practices across dimensions

Multivariate logistic regression demonstrated that holding a master’s degree or higher [aOR = 3.659, 95% CI: (1.309, 10.225), *p* = 0.013] was independently associated with higher knowledge scores (Supplementary Table S1). Additionally, a higher knowledge score [aOR = 1.085, 95% CI: (1.016, 1.158), *p* = 0.015] was independently associated with a positive attitude (Supplementary Table S2). Furthermore, a higher attitude score [aOR = 1.209, 95% CI: (1.164, 1.256), *p* < 0.001] was independently associated with more proactive practices (Supplementary Table S3).

### Path analysis

**Figure 1:**
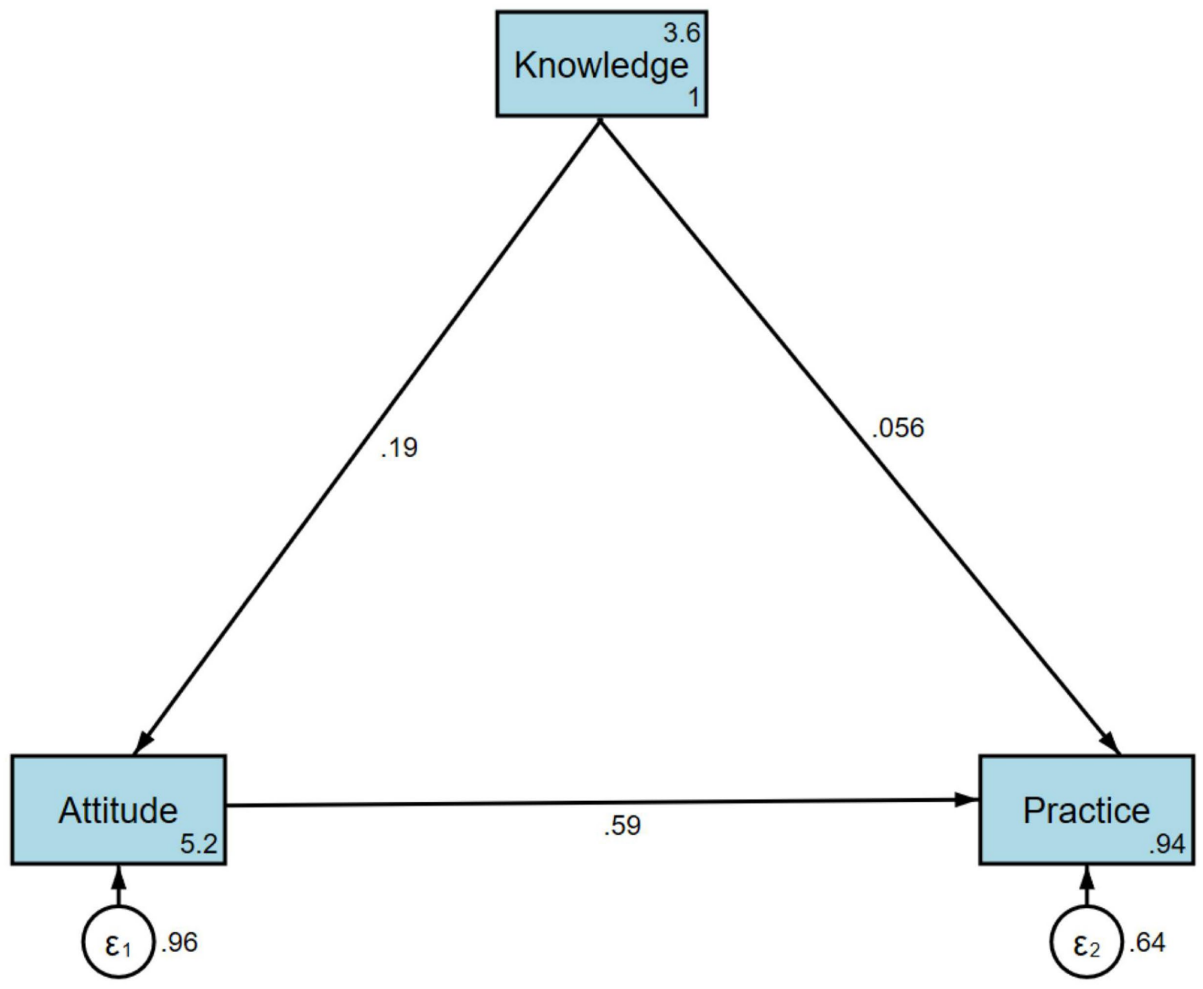
The structural equation model (SEM). Rectangle shows observed variables, ellipses indicate potential variables, and circles represent residual terms.

The path analysis revealed that knowledge directly influenced attitude (*β* = 0.191, *p* < 0.001), and attitude had a direct effect on practice (*β* = 0.591, *p* < 0.001). Additionally, knowledge indirectly affected practice through attitude (*β* = 0.113, *p* < 0.001) (Supplementary Table S4; Figure 1).

## Discussion

Patients with hand eczema in Guangdong, China, exhibited insufficient knowledge but maintained positive attitudes and relatively inactive practices toward managing their condition. Clinically, targeted educational interventions focusing on enhancing patient knowledge are recommended, as improving knowledge may lead to more proactive practices and better management of hand eczema.

Many patients feel they lack sufficient knowledge regarding the causes of eczema and how best to treat it (17). Moreover, public knowledge of hand eczema is generally low and often influenced by misconceptions, leading to misunderstanding and stigmatization of patients with the condition (18). Although most participants recognized the importance of using moisturizers as a preventive measure (as reflected in the attitude dimension), there is a lack of understanding in the knowledge and behavior dimensions regarding when to use moisturizing cream, how to use it, and which type of moisturizing cream to use. For instance, Participants are not aware of whether moisturizer should be applied multiple times in humid environments. Additionally, over 50% of participants do not use fragrance-free moisturizers, and they fail to apply the moisturizer evenly across their entire hands. This knowledge gap can easily impede effective self-management by patients.

Knowledge and attitude were correlated, as were attitude and practice, indicating an association between knowledge and attitudes. However, the direct effect of knowledge on practice was weaker, highlighting that knowledge alone is insufficient to translate into proactive behavior changes. This finding is consistent with the KAP theory, where changes in practice typically follow the development of attitudes (19, 20). The stronger association between attitude and practice, both in SEM and multivariate analysis, suggests that interventions should focus not only on improving knowledge but also on reinforcing positive attitudes to encourage proactive behaviors. Previous research has shown that improved knowledge and attitudes can promote better self-management behaviors, leading to lower disease severity and improved quality of life in patients with atopic dermatitis, supporting our hypothesis that strengthening both knowledge and attitudes may ultimately improve clinical severity (HECSI) and life quality (DLQI) in hand eczema (21). In addition, although we noted a strong association between attitudes and behaviors, patients’ positive attitudes but less proactive practices suggest that patient practice may be influenced by barriers other than attitudes. Environmental factors, such as occupational requirements for frequent hand washing among healthcare workers, may force compromises in skin care practices. Additionally, stigma associated with public skin care routines (such as applying moisturizers at work) may inhibit practice implementation despite positive attitudes.

The analysis revealed significant differences in knowledge and practice across several demographic variables, particularly education level, age, and place of residence. Patients with higher education levels, especially those with a master’s degree or above, demonstrated better knowledge and more proactive practices, consistent with previous studies showing that higher health literacy promotes better disease management. Younger patients tended to engage more actively in preventive behaviors, such as regular moisturizing and avoiding irritants, possibly due to greater exposure to health information through digital media and higher adaptability to lifestyle adjustments. In addition, urban residents showed higher knowledge and practice scores than rural residents, likely reflecting better access to dermatological care and health education resources. These findings suggest that socioeconomic and educational disparities influence patients’ self-management capacities and should be considered when designing targeted educational interventions (22, 23).

The analysis of the knowledge dimension revealed that while most participants indicated a reasonable understanding of hand eczema as an inflammatory skin condition, significant gaps existed in key areas. Many respondents were unclear about the role of moisturizers, the appropriate use of gloves, and the importance of patch testing for allergens. The lack of awareness regarding the proper use of gloves and moisturizers suggests that patients may not be receiving adequate information from healthcare providers or may not fully understand the educational materials provided to them. To address these gaps, it is essential to develop targeted educational interventions that focus on practical, actionable knowledge, such as how to properly use moisturizers and the role of gloves in preventing exacerbations of hand eczema (24–26). These interventions could be tailored to individuals with lower educational backgrounds and those in high-risk occupations, where the use of gloves and exposure to irritants is more prevalent.

In the attitude dimension, most participants indicated generally positive attitudes toward managing hand eczema, with a majority agreeing on the importance of using moisturizers and avoiding direct contact with irritants such as chemical products. However, when examining areas of disagreement, certain preventive measures, such as changing jobs to alleviate severe hand eczema and avoiding frequent hand washing, were met with notable resistance. This indicates that while participants generally acknowledged the significance of moisturizers and other preventive practices, measures perceived as impractical or disruptive to daily life were less likely to be accepted. This highlights the need for tailored education and practical solutions that align with participants’ lifestyles and occupational demands. Future research should explore effective ways to enhance public awareness and acceptance of preventive practices, as well as develop policies and workplace accommodations that support individuals in managing hand eczema without significantly impacting their daily routines (27–30).

In the practice dimension, the findings were less encouraging. A significant proportion of participants failed to adopt essential management practices, such as regularly applying moisturizers, avoiding irritants, and using protective gloves in wet environments. This behavioral gap may stem from a combination of psychological barriers (e.g., low self-efficacy), environmental or economic constraints, and a lack of personalized, effective patient education (31). To address this, interventions should not only focus on educating patients about proper management techniques but also on providing practical solutions, such as offering affordable, fragrance-free skincare products and recommending specific types of gloves that are both protective and comfortable for long-term use. Additionally, healthcare providers should work closely with patients in high-risk occupations to develop customized management plans that account for the specific challenges they face in their work environments. Furthermore, incorporating reminders via text messages or social media platforms could help increase the frequency of moisturizer use, reinforcing good skincare habits and improving treatment outcomes (32–35).

This study has several limitations. First, the use of a self-reported questionnaire may have introduced response bias, as participants might overestimate or misreport their knowledge and practices. Second, the cross-sectional design prevents us from establishing causal relationships between knowledge, attitudes, and practices. Finally, the study was conducted at a single hospital in Guangdong, limiting the generalizability of the findings to other regions or healthcare settings. Additionally, as the study was conducted in a hospital setting, there was an overrepresentation of moderate-to-severe cases (87.8% of participants), which may not reflect the full spectrum of hand eczema severity in the general population. This suggests that our findings might be more applicable to patients with more severe disease, and future studies should consider including patients from community settings to capture milder cases.

In conclusion, patients with hand eczema in Guangdong, China, exhibited inadequate knowledge about their condition, while maintaining generally positive attitudes, though their practices were less proactive. Our findings suggest that targeted educational interventions focusing on knowledge enhancement may be valuable for improving self-management behaviors. While our cross-sectional design prevents causal inference, the observed pathway from knowledge to practice suggests that targeted education may potentially translate into better clinical management, offering a rationale for future interventional studies to verify improvements in HECSI and DLQI scores. Although this study is a cross-sectional design and, therefore, cannot establish a cause-and-effect relationship (causality), the KAP model path analysis suggests that improved knowledge is associated with more proactive practice behaviors. We reasonably hypothesize that this improvement in practice (such as increased frequency of moisturization and correct use of gloves) is a prerequisite for improving clinical symptoms and enhancing quality of life. Therefore, validating whether educational interventions targeting the knowledge gaps (as revealed by our data) can lead to actual improvements in HECSI and DLQI scores is a critical direction for future longitudinal studies or clinical trials.

## Data Availability

The original contributions presented in the study are included in the article/Supplementary material, further inquiries can be directed to the corresponding author.

## Supplementary material

SUPPLEMENTARY FIGURE S1 Structural equation model for knowledge, attitude, and practice constructs.
