# Supplementary material for: Knowledge, attitudes, and practices of hand eczema patients in Guangdong, China
Source: Front Public Health. 2025 Dec 18;13:1706796. doi: 10.3389/fpubh.2025.1706796 (PMC12756390; doi:10.3389/fpubh.2025.1706796)
Supplement: SUPPLEMENTARY FIGURE S1 — Structural equation model for knowledge, attitude, and practice constructs. [file Data_Sheet_1.docx]

The knowledge section consists of 12 questions, with 10 single-choice items scored 1 points for a correct response and 0 point for an incorrect one. Two multiple-choice questions (K5 and K6) allow for multiple correct answers, awarding 1 points per correct choice, with K5 offering a maximum of 7 points and K6 a maximum of 6 points. An incorrect choice in these multiple-choice questions results in 0 point for the entire question. The total score for the knowledge section ranges from 0 to 23 points. The attitude section contains 10 questions using a five-point Likert scale, with responses ranging from very positive (5 points) to very negative (1 points), yielding a score range of 10 to 50 points. Similarly, the practice section includes 8 questions, also rated on a five-point Likert scale, ranging from very positive (5 points) to very negative (1 points), with a total score range of 8 to 40 points.

**Survey on Knowledge, Attitudes, and Practices of Hand Eczema Patients in Guangdong, China**

Hello!

We would like to invite you to participate in our study. The purpose of this study is to investigate the knowledge, attitudes, and practices of hand eczema patients in Guangdong.

The survey will be anonymous, so your information will not be disclosed, and the results will be used for academic research only, so please feel free to fill in the survey according to your actual situation.

Thank you for your cooperation and support!

□I am a hand eczema patients.

□I am informed and agree that the data collected will be used for scientific research.

Rating form number:: __________________

## Part I Basic Information

**1. Your gender:**

a.Male b.Female

**2. Your age: _______years old.**

**3. Your education:**

a.Middle school and below b.High school/technical school c.College/bachelor’s degree d.Master’s degree and above

**4.Residence:**

a.Urban b.Rural

**5.Occupation:**

a.Healthcare worker b.Beauty and hairdressing industry c.Housewife d.Cleaning and janitorial work e.Hardware/construction industry f.Food service industry g.Clerical staff h.Student i.Other, please fill in______

**6. Your years of employment:**

a.≤1 year b.1-2 years c.2-5 years d.＞5 years

**7. Duration of hand eczema:**

a.≤3 months b.3 months -1 year c.1-2 years d.2-5 years e.＞5 years

**8. Dermatology Life Quality Index (DLQI) Score: ________.**

**9. Hand Eczema Severity Index (HECSI) Score: ________.**

**10. Have you or your immediate family members ever had a history of atopic diseases? (Such as allergic rhinitis, allergic asthma, allergic conjunctivitis, atopic dermatitis/eczema etc.)**

a.Yes b.No c.Not sure

**11. Have you ever undergone a patch test (allergen test): ________.**

a.Yes b.No c.Not sure

## Part II Knowledge Dimension

**1. Hand eczema is an inflammatory skin disease that occurs on the hands.**

a.True b.False c.Not sure

**2. Hand eczema is always accompanied by itching.**

a.True b.False c.Not sure

**3. Hand eczema is characterized by diverse clinical manifestations.**

a.True b.False c.Not sure

**4. Individuals with an atopic constitution (allergic constitution) have a significantly higher risk of developing hand eczema compared to those without an atopic constitution.**

a.True b.False c.Not sure

**5. The etiology of hand eczema is complex. External causes may include: (Multiple choices allowed)**

a.Metal products (nickel, chromium, cobalt)

b.Natural rubber

c.Fragrances

d.Food proteins (raw meat, animal organs, grains)

e.Strong irritants such as acids, alkalis, organic solvents, or other chemical products

f.Weak irritants such as water, soap, detergents, oil, printing ink

g.Mechanical damage, such as trauma, scratching, prolonged friction

h.None of the above

**6. The etiology of hand eczema is complex. Internal causes may include: (Multiple choices allowed)**

a.Genetic factors b.Atopic constitution c.Mental state d.Hormonal levels e.Immune status of the body f.Changes in trace elements g.None of the above

**7. To identify potential allergens, all patients with hand eczema undergo patch testing (allergen testing).**

a.True b.False c.Not sure

**8. Damage to the skin barrier function is the central factor in the recurrence of hand eczema.**

a.True b.False c.Not sure

**9. When washing hands, patients with hand eczema should avoid using soaps or other cleansers. Instead, they should rinse their hands with warm water, thoroughly dry them, and promptly moisturize.**

a.True b.False c.Not sure

**10. Topical corticosteroids are the first choice of treatment for patients with hand eczema (e.g., hydrocortisone cream, desonide cream, mometasone furoate cream).**

a.True b.False c.Not sure

**11. Patients with hand eczema should preferably use rubber gloves (nitrile gloves) and film gloves (vinyl gloves).**

a.True b.False c.Not sure

**12. If gloves are not worn, moisturizers should be applied frequently when working in wet environments or with wet hands.**

a.True b.False c.Not sure

## Part III Attitude Dimension

**1. Frequent handwashing should be avoided.**

a.Strongly agree b.Agree c.Neutral d.Disagree e.Strongly disagree

**2. Direct contact with chemical products such as hand sanitizers, laundry detergents, and alcohol should be avoided.**

a.Strongly agree b.Agree c.Neutral d.Disagree e.Strongly disagree

**3. Moisturizers should be used frequently and consistently.**

a.Strongly agree b.Agree c.Neutral d.Disagree e.Strongly disagree

**4. Protective gloves should be worn when working in wet environments.**

a.Strongly agree b.Agree c.Neutral d.Disagree e.Strongly disagree

**5. The material and usage of gloves are crucial.**

a.Strongly agree b.Agree c.Neutral d.Disagree e.Strongly disagree

**6. Applying moisturizer in addition to using gloves can enhance protective effects.**

a.Strongly agree b.Agree c.Neutral d.Disagree e.Strongly disagree

**7. Wearing rings and other hand jewelry negatively impacts eczema.**

a.Strongly agree b.Agree c.Neutral d.Disagree e.Strongly disagree

**8. Avoid vigorous scratching or rubbing of the hands.**

a.Strongly agree b.Agree c.Neutral d.Disagree e.Strongly disagree

**9. Actively identifying and eliminating any potential causes and aggravating factors is important for treating hand eczema.**

a.Strongly agree b.Agree c.Neutral d.Disagree e.Strongly disagree

**10. Changing jobs can alleviate severe cases of hand eczema.**

a.Strongly agree b.Agree c.Neutral d.Disagree e.Strongly disagree

## Part IV Practice Dimension

**1. Use fragrance-free topical skincare and moisturizing products (e.g., hand cream).**

a. Always b. Often c. Sometimes d. Occasionally e. Never

**2. Do not wear rings or other jewelry when working with wet hands.**

a. Always b. Often c. Sometimes d. Occasionally e. Never

**3. When wearing occlusive gloves for more than 10 minutes, wear cotton gloves underneath.**

a. Always b. Often c. Sometimes d. Occasionally e. Never

**4. Apply moisturizer multiple times after finishing work each day and before bedtime.**

a. Always b. Often c. Sometimes d. Occasionally e. Never

**5. Do not reuse disposable gloves.**

a. Always b. Often c. Sometimes d. Occasionally e. Never

**6. Wash hands with cold/warm water, avoiding hot water.**

a. Always b. Often c. Sometimes d. Occasionally e. Never

**7. Evenly apply moisturizer to the entire hand, including fingertips, between fingers, and the back of the hands.**

a. Always b. Often c. Sometimes d. Occasionally e. Never

**8. Wear gloves outdoors in cool weather to protect your hands from drying out.**

a. Always b. Often c. Sometimes d. Occasionally e. Never
