# Supplementary material for: Knowledge, attitudes, and practices of hand eczema patients in Guangdong, China
Source: Front Public Health. 2025 Dec 18;13:1706796. doi: 10.3389/fpubh.2025.1706796 (PMC12756390; doi:10.3389/fpubh.2025.1706796)
Supplement: Supplementary file 2 [file Data_Sheet_2.docx]

**Table S1. Univariate and multivariate analysis for knowledge dimension**

| **Knowledge** | **Univariate analysis** |  | **Multivariate analysis** |  |
| --- | --- | --- | --- | --- |
|  | **aOR(95%CI)** | **P** | **aOR(95%CI)** | **P** |
| **Gender** |  |  |  |  |
| Male |  |  |  |  |
| Female | 1.217 (0.857,1.725) | 0.270 |  |  |
| **Age** |  |  |  |  |
| 30 years old or below |  |  |  |  |
| 31-35 years old | 0.855 (0.513,1.414) | 0.542 | 0.799 (0.479,1.332) | 0.389 |
| 36-40 years old | 0.825 (0.498,1.359) | 0.453 | 0.812 (0.490,1.344) | 0.418 |
| 40 years old or above | 0.635 (0.387,1.030) | 0.068 | 0.677 (0.411,1.114) | 0.124 |
| **Education** |  |  |  |  |
| Middle school and below |  |  |  |  |
| High school/technical school | 1.773 (0.957,3.298) | 0.069 | 1.519 (0.778,2.965) | 0.221 |
| College/bachelor’s degree | 2.056 (1.153,3.679) | 0.014 | 1.748 (0.924,3.309) | 0.086 |
| Master’s degree and above | 4.154 (1.609,11.848) | 0.005 | 3.659 (1.309,10.225) | **0.013** |
| **Residence** |  |  |  |  |
| Urban |  |  |  |  |
| Rural | 0.692 (0.448,1.075) | 0.099 | 0.839 (0.520,1.353) | 0.470 |
| **Years of employment** |  |  |  |  |
| ≤2 years |  |  |  |  |
| 2-5 years | 1.026 (0.559,1.866) | 0.935 |  |  |
| ＞5 years | 0.877 (0.523,1.443) | 0.610 |  |  |
| **Duration of Hand Eczema** |  |  |  |  |
| ≤3 months |  |  |  |  |
| 3 month-1 year | 0.945 (0.622,1.431) | 0.790 |  |  |
| 1-2 years | 1.093 (0.664,1.807) | 0.727 |  |  |
| ＞2 years | 0.901 (0.531,1.538) | 0.701 |  |  |
| **Dermatology Life Quality Index (DLQI) Score** |  |  |  |  |
| No or mild impact (0-5 points) |  |  |  |  |
| Moderate impact (6-10 points) | 0.879 (0.563,1.371) | 0.570 |  |  |
| Severe impact (11-20 points) | 1.352 (0.890,2.053) | 0.157 |  |  |
| Very severe impact (21-30 points) | 1.068 (0.530,2.214) | 0.856 |  |  |
| **Hand Eczema Severity Index (HECSI) Score** |  |  |  |  |
| Almost clear, 1-16 |  |  |  |  |
| Moderate, 17-37 | 0.950 (0.559,1.593) | 0.848 |  |  |
| Severe, 38-116 | 1.230 (0.711,2.105) | 0.453 |  |  |
| **Have you or your immediate family members ever had a history of atopic diseases?** |  |  |  |  |
| Yes |  |  |  |  |
| No | 0.819 (0.447,1.468) | 0.508 |  |  |
| Not sure | 1.295 (0.707,2.324) | 0.393 |  |  |
| **Have you ever undergone a patch test (allergen test)?** |  |  |  |  |
| Yes |  |  |  |  |
| No | 0.726 (0.387,1.313) | 0.301 |  |  |
| Not sure | 1.098 (0.548,2.149) | 0.787 |  |  |

**Table S2. Univariate and multivariate analysis for attitude dimension**

| **Attitude** | **Univariate analysis** |  | **Multivariate analysis** |  |
| --- | --- | --- | --- | --- |
|  | **aOR(95%CI)** | **P** | **aOR(95%CI)** | **P** |
| **Knowledge** | 1.097 (1.028,1.170) | 0.005 | 1.085 (1.016,1.158) | 0.015 |
| **Gender** |  |  |  |  |
| Male |  |  |  |  |
| Female | 0.791 (0.562,1.109) | 0.174 |  |  |
| **Age** |  |  |  |  |
| 30 years old or below |  |  |  |  |
| 31-35 years old | 1.255 (0.781,2.020) | 0.349 |  |  |
| 36-40 years old | 1.204 (0.752,1.931) | 0.440 |  |  |
| 40 years old or above | 1.091 (0.686,1.736) | 0.714 |  |  |
| **Education** |  |  |  |  |
| Middle school and below |  |  |  |  |
| High school/technical school | 1.571 (0.848,2.964) | 0.155 | 1.249 (0.642,2.430) | 0.513 |
| College/bachelor’s degree | 1.721 (0.965,3.131) | 0.069 | 1.332 (0.705,2.516) | 0.377 |
| Master’s degree and above | 2.245 (0.944,5.477) | 0.070 | 1.697 (0.681,4.229) | 0.256 |
| **Residence** |  |  |  |  |
| Urban |  |  |  |  |
| Rural | 0.668 (0.431,1.030) | 0.069 | 0.765 (0.479,1.222) | 0.262 |
| **Years of employment** |  |  |  |  |
| ≤2 years |  |  |  |  |
| 2-5 years | 1.322 (0.747,2.344) | 0.338 |  |  |
| ＞5 years | 1.006 (0.621,1.631) | 0.981 |  |  |
| **Duration of Hand Eczema** |  |  |  |  |
| ≤3 months |  |  |  |  |
| 3 month-1 year | 1.142 (0.765,1.706) | 0.515 |  |  |
| 1-2 years | 0.927 (0.576,1.491) | 0.755 |  |  |
| ＞2 years | 0.819 (0.489,1.369) | 0.447 |  |  |
| **Dermatology Life Quality Index (DLQI) Score** |  |  |  |  |
| No or mild impact (0-5 points) |  |  |  |  |
| Moderate impact (6-10 points) | 0.914 (0.591,1.414) | 0.687 |  |  |
| Severe impact (11-20 points) | 0.793 (0.532,1.182) | 0.255 |  |  |
| Very severe impact (21-30 points) | 0.996 (0.501,1.996) | 0.992 |  |  |
| **Hand Eczema Severity Index (HECSI) Score** |  |  |  |  |
| Almost clear, 1-16 |  |  |  |  |
| Moderate, 17-37 | 0.749 (0.447,1.245) | 0.267 |  |  |
| Severe, 38-116 | 0.732 (0.431,1.232) | 0.243 |  |  |
| **Have you or your immediate family members ever had a history of atopic diseases?** |  |  |  |  |
| Yes |  |  |  |  |
| No | 1.389 (0.783,2.483) | 0.262 |  |  |
| Not sure | 1.152 (0.653,2.048) | 0.626 |  |  |
| **Have you ever undergone a patch test (allergen test)?** |  |  |  |  |
| Yes |  |  |  |  |
| No | 0.701 (0.390,1.240) | 0.226 |  |  |
| Not sure | 0.659 (0.345,1.239) | 0.198 |  |  |

**Table S3. Univariate and** **multivariate analysis for practice dimension**

| **Practice** | **Univariate analysis** |  | **Multivariate analysis** |  |
| --- | --- | --- | --- | --- |
|  | **aOR(95%CI)** | **P** | **aOR(95%CI)** | **P** |
| **Knowledge** | 1.086 (1.017,1.160) | 0.013 | 1.008 (0.934,1.087) | 0.846 |
| **Attitude** | 1.207 (1.163,1.253) | <0.001 | 1.209 (1.164,1.256) | <0.001 |
| **Gender** |  |  |  |  |
| Male |  |  |  |  |
| Female | 0.808 (0.570,1.140) | 0.226 |  |  |
| **Age** |  |  |  |  |
| 30 years old or below |  |  |  |  |
| 31-35 years old | 1.283 (0.789,2.088) | 0.316 |  |  |
| 36-40 years old | 0.982 (0.609,1.582) | 0.942 |  |  |
| 40 years old or above | 0.835 (0.522,1.332) | 0.450 |  |  |
| **Education** |  |  |  |  |
| Middle school and below |  |  |  |  |
| High school/technical school | 2.455 (1.322,4.632) | 0.005 | 1.759 (0.829,3.732) | 0.141 |
| College/bachelor’s degree | 2.052 (1.152,3.716) | 0.016 | 1.490 (0.730,3.038) | 0.273 |
| Master’s degree and above | 2.350 (0.986,5.772) | 0.057 | 1.566 (0.568,4.321) | 0.386 |
| **Residence** |  |  |  |  |
| Urban |  |  |  |  |
| Rural | 0.621 (0.403,0.957) | 0.031 | 0.744 (0.439,1.261) | 0.273 |
| **Years of employment** |  |  |  |  |
| ≤2 years |  |  |  |  |
| 2-5 years | 1.060 (0.598,1.877) | 0.840 |  |  |
| ＞5 years | 1.343 (0.825,2.178) | 0.232 |  |  |
| **Duration of Hand Eczema** |  |  |  |  |
| ≤3 months |  |  |  |  |
| 3 month-1 year | 0.956 (0.635,1.436) | 0.830 |  |  |
| 1-2 years | 0.803 (0.496,1.297) | 0.369 |  |  |
| ＞2 years | 0.947 (0.563,1.601) | 0.839 |  |  |
| **Dermatology Life Quality Index (DLQI) Score** |  |  |  |  |
| No or mild impact (0-5 points) |  |  |  |  |
| Moderate impact (6-10 points) | 1.027 (0.660,1.599) | 0.906 |  |  |
| Severe impact (11-20 points) | 1.025 (0.683,1.536) | 0.904 |  |  |
| Very severe impact (21-30 points) | 0.606 (0.302,1.208) | 0.155 |  |  |
| **Hand Eczema Severity Index (HECSI) Score** |  |  |  |  |
| Almost clear, 1-16 |  |  |  |  |
| Moderate, 17-37 | 1.076 (0.645,1.788) | 0.777 |  |  |
| Severe, 38-116 | 1.345 (0.793,2.273) | 0.268 |  |  |
| **Have you or your immediate family members ever had a history of atopic diseases?** |  |  |  |  |
| Yes |  |  |  |  |
| No | 1.284 (0.722,2.285) | 0.394 | 0.989 (0.514,1.904) | 0.975 |
| Not sure | 1.748 (0.987,3.104) | 0.055 | 1.489 (0.778,2.847) | 0.229 |
| **Have you ever undergone a patch test (allergen test)?** |  |  |  |  |
| Yes |  |  |  |  |
| No | 0.648 (0.350,1.164) | 0.156 |  |  |
| Not sure | 0.787 (0.401,1.510) | 0.477 |  |  |

**Table S4. Analysis of direct and indirect effects**

| **Model paths** | | **Total effects** | | **Direct Effect** | | **Indirect effect** | |
| --- | --- | --- | --- | --- | --- | --- | --- |
|  |  | **β(95%CI)** | **P** | **β(95%CI)** | **P** | **β(95%CI)** | **P** |
| Asum |  |  |  |  |  |  |  |
|  | Ksum | 0.191 (0.115,0.266) | <0.001 | 0.191 (0.115,0.266) | <0.001 |  |  |
| Psum |  |  |  |  |  |  |  |
|  | Asum | 0.591 (0.538,0.643) | <0.001 | 0.591 (0.538,0.643) | <0.001 |  |  |
|  | Ksum | 0.169 (0.093,0.246) | <0.001 | 0.056 (-0.008,0.121) | 0.085 | 0.113 (0.067,0.159) | <0.001 |
