# Supplementary figures and images for: Knowledge, attitudes, and practices of hand eczema patients in Guangdong, China
Source: Front Public Health. 2025 Dec 18;13:1706796. doi: 10.3389/fpubh.2025.1706796 (PMC12756390; doi:10.3389/fpubh.2025.1706796)

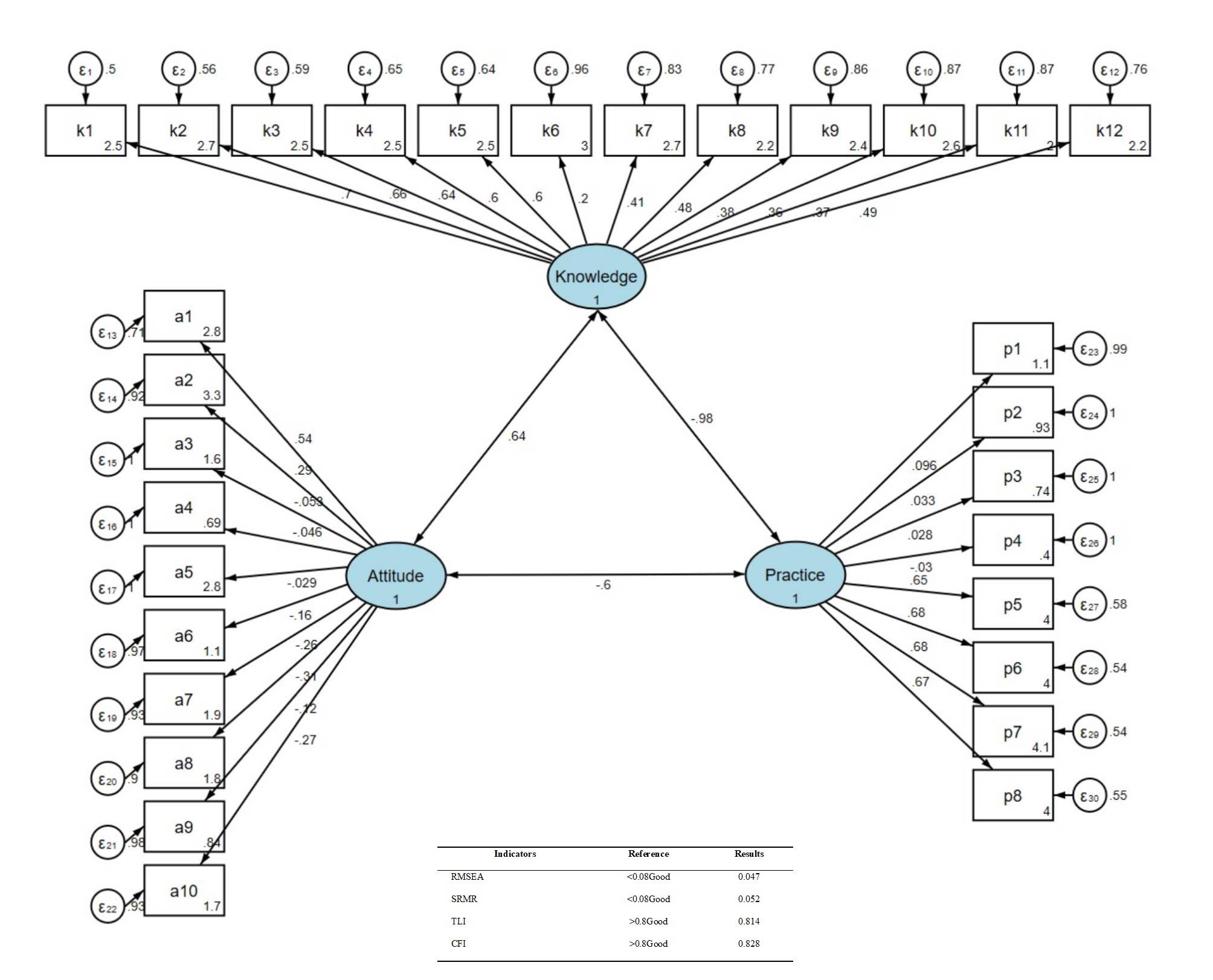

Supplement: Supplementary file 3 [file Image_1.jpeg]
